# Idiopathic myelitis presenting as Brown-Séquard syndrome: two case reports and a review of the literature

**DOI:** 10.1186/s13256-021-02834-1

**Published:** 2021-05-12

**Authors:** Xi Peng, Liang Wang

**Affiliations:** 1 Department of Neurology, The Second Affiliated Hospital of Chongqing Medical University, Chongqing, 400010 China; 2 Department of Neurology, The First Affiliated Hospital of Chongqing Medical University, Chongqing, 400016 China

**Keywords:** Brown-Séquard syndrome, Myelitis, Differential diagnosis

## Abstract

**Background:**

Brown-Séquard syndrome often occurs in spinal cord injury, and few myelitis patients present with Brown-Séquard syndrome.

**Case presentation:**

A 33-year-old Han man was admitted with neck pain plus numbness in the right limbs for 2 days and weakness in the left limbs for 1 day. Examination was significant for left limbs with grade 4 muscle power, positive left Babinski sign, diminished vibration sensation in the left limbs and decreased pain below the right clavicle dermatome. The cerebrospinal fluid (CSF) cell count was 24 × 10^6^/L, and the protein count was 185 mg/L. Cervical magnetic resonance imaging (MRI) indicated abnormal swelling signals in the medulla-cervical cord long segment and enhanced signals in the C2-3 region. In the second case, a 47-year-old Han woman was admitted with weakness in the right lower limb and numbness in the left lower limb for more than 20 days. Examination was significant for the right lower limb with grade 4 muscle power, left knee hyperreflexia, positive left Babinski sign, diminished vibration sensation in the right lower limb and decreased pain below the right T2 dermatome. Cervical MRI indicated hyperintense and enhanced signals in the C7-T2 region. In these two cases, CSF culture, oligoclonal band (OB) and aquaporin 4 (AQP4) antibody were negative. Brain MRI was normal. Their symptoms and MRI results improved after treatment with methylprednisolone.

**Conclusions:**

Myelitis can present as Brown-Séquard syndrome, providing an extended reference in terms of the differential diagnosis for clinical physicians.

## Background

Brown-Séquard syndrome describes a lesion involving only one side of the spinal cord, which is characterized by ipsilateral impaired proprioception, vibratory sensation and motor function, and contralateral loss of pain and temperature sensation [1]. It mostly occurs in spinal cord injury, extramedullary spinal cord tumors and spinal hemorrhages, and sometimes it is caused by demyelinating diseases such as multiple sclerosis [1]. However, few myelitis patients present with Brown-Séquard syndrome. We describe two cases of myelitis presenting as Brown-Séquard syndrome.

## Case presentation

### Case 1

**Fig. 1:**
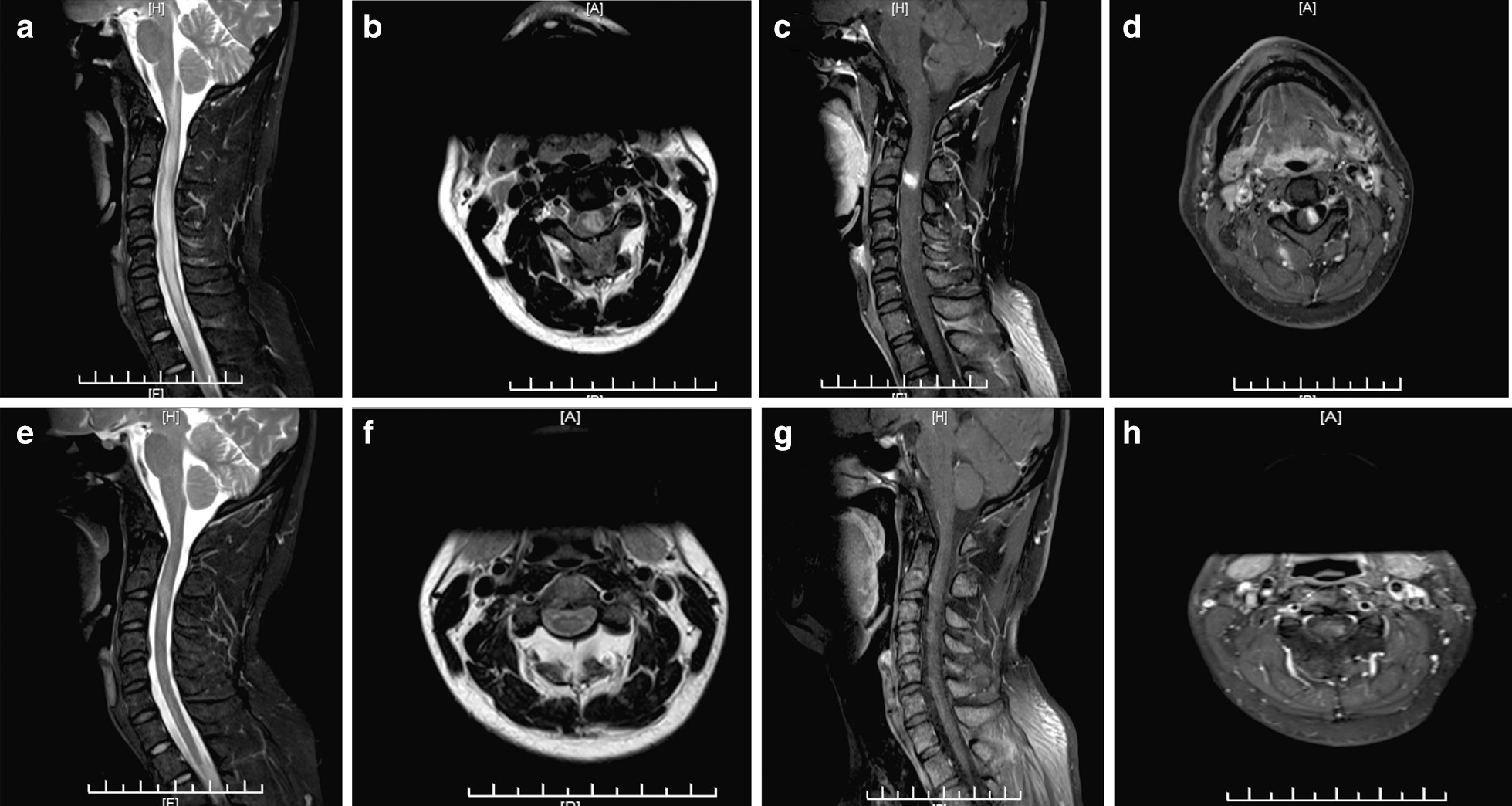
**a**–**d** Cervical magnetic resonance imaging (MRI) shows hyperintense signal changes from the medulla to the cervical cord with long segments in the T2-weighted image and enhanced signals from C2 to C3 in the T1-weighted image: **a** midsagittal T2-weighted image, **b** axial T2-weighted image, **c** midsagittal contrast-enhanced T1-weighted image, **d** axial contrast-enhanced T1-weighted image. **e**–**h** After treatment, cervical MRI shows that the swelling was reduced and that there were fewer enhanced lesions in the C2-3 region

A 33-year-old Han man was admitted with neck pain plus numbness in the right upper and lower limbs for 2 days. Two days earlier, the patient had a cold and presented with neck pain, especially when turning his neck. Numbness was present in the right upper and lower limbs and was progressive. One day earlier, the patient presented with weakness in the left upper and lower limbs, which did not negatively affect his daily movement. No fever, headache, visual impairment or defecation disorders were observed. No abnormality was observed in the past medical history. Physical examination revealed the following: Vital signs were stable. Cranial nerves were normal. Muscle power of the left upper and lower limbs was grade 4. Muscle tone was normal and reflexes were normal in all four limbs. Pain was decreased below the level of the right clavicle, and proprioception and vibration sensation in the left upper and lower limbs were diminished. The left Babinski sign was positive. The results of a routine blood test, hepatorenal function examination, electrolyte examination and antinuclear antibody spectrum were normal. The cancer spectrum was negative. Serum herpes, *Cytomegalovirus* (CMV) and Epstein-Barr virus (EBV) deoxyribonucleic acid (DNA) levels were negative. The lumbar puncture cerebrospinal fluid (CSF) cell count was 24 × 10^6^/L, and the protein count was 185 mg/L. CSF smear and acid-fast staining were negative. CSF culture, oligoclonal band (OB) and aquaporin 4 (AQP4) antibody were negative. Visual evoked potential (VEP) indicated normality in the P100 incubation period. Brain magnetic resonance imaging (MRI) was normal. Cervical MRI indicated abnormal swelling signals in the medulla-cervical cord long segment and enhanced signals in the C2-3 region (Fig. 1a–d). Myelitis was considered, and neuromyelitis optica spectrum disorders (NMOSD) with negative AQP4 antibody was not excluded. The patient was administered methylprednisolone (1000 mg for 5 days) and then prednisone (65 mg, orally, once a day, reduced by 10 mg per week). On the third day after methylprednisolone pulse therapy was administered, the symptoms improved significantly, and they were completely resolved after 14 days. Cervical MRI was rechecked and indicated that the swelling had been significantly reduced and that the enhanced lesions in the C2-3 region had shrunk (Fig. 1e–h).

### Case 2

**Fig. 2:**
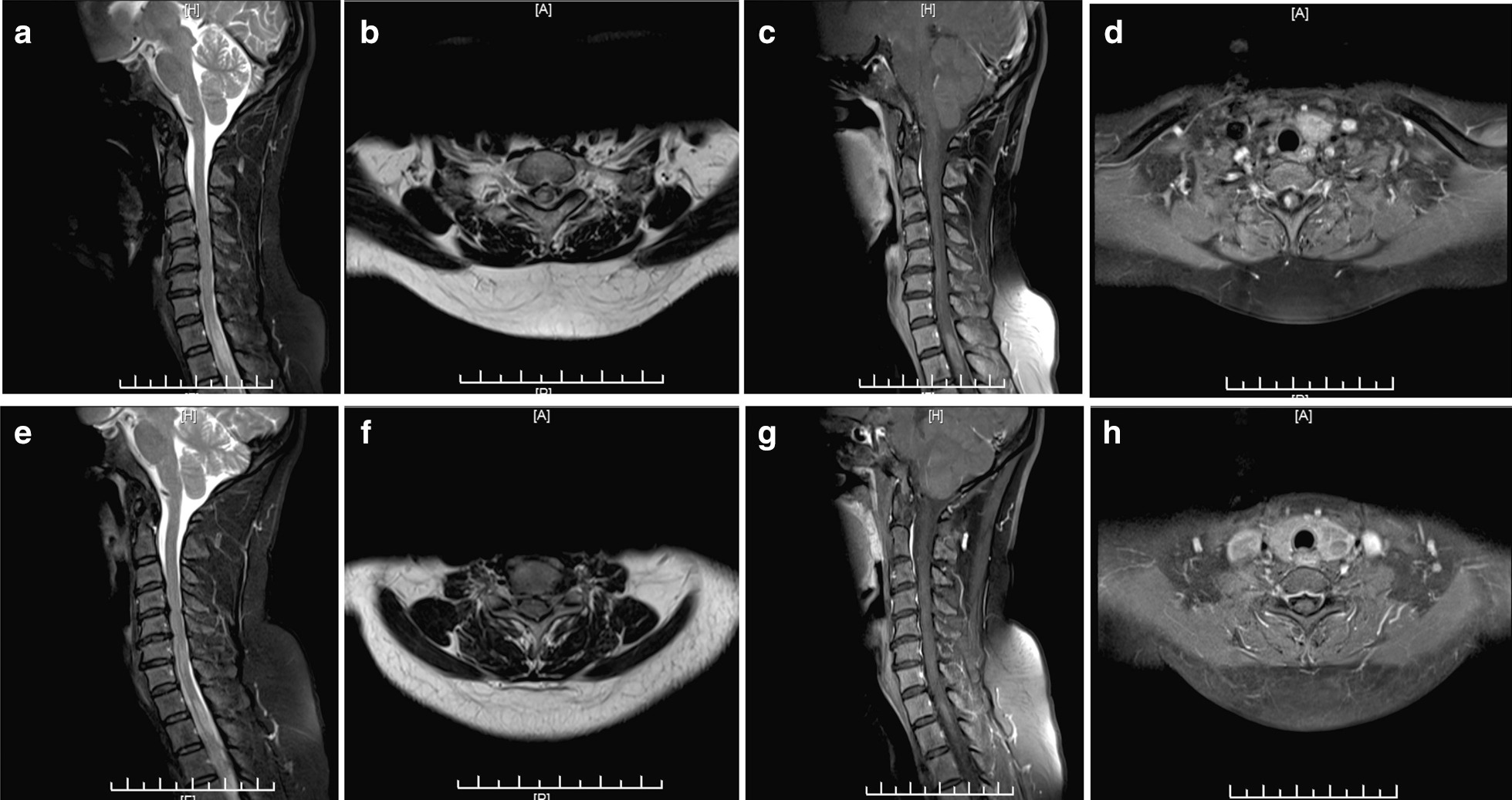
**a**–**d** Cervical magnetic resonance imaging (MRI) shows hyperintense signal changes from C7 to T2 in the T2-weighted image and shows the gadolinium-enhanced lesion in the T1-weighted image. **e**–**h** After treatment, cervical MRI shows that the swelling was reduced and that there were fewer enhanced lesions in the C7-T2 region

A 47-year-old Han woman was admitted with weakness in the right lower limb and numbness in the left lower limb for more than 20 days. More than 20 days earlier, the patient presented with weakness in the right lower limb without cause. Half a day later, numbness was observed in the left lower limb accompanied by a cold sensation. No defecation disorders were observed. Lumbar MRI at an external hospital indicated protrusion of the intervertebral disk in the S3-4 and S4-5 regions. The patient did not improve after lumbar disease was treated and had aggravated weakness in the right lower limb and walking difficulties. No abnormalities were observed in the past medical history. Physical examination: Vital signs were stable. Cranial nerves were normal. Grade 4 muscle power was found in the right lower limb, while grade 5 muscle power was revealed in the left lower limb. Normal muscle tone was found in all four limbs. The knee reflex was exaggerated in her right lower limb. Pain was reduced below the left sternal angle. Proprioception and vibration sensation in the right lower limb were diminished. The right Babinski sign was positive. The results of a routine blood test, hepatorenal function examination, electrolyte examination and antinuclear antibody spectrum were normal. Serum herpes, CMV and EBV DNA levels were negative. The CSF cell count was 1 × 10^6^/L, and the protein level was 328 mg/L. CSF smear and acid-fast staining were negative. CSF culture, OB and AQP4 antibody were negative. Brain MRI was normal. Cervical MRI indicated hyperintense and enhanced signals in the C7-T2 region (Fig. 2a–d). Myelitis was considered. The patient was administered methylprednisolone (1000 mg for 5 days) and then prednisone (55 mg, orally, once a day, reduced by 10 g per week). After methylprednisolone pulse therapy was given, the symptoms were significantly improved. Fifteen days later, cervical MRI was rechecked, indicating that the lesions were significantly reduced (Fig. 2e–h). The patient recovered and was discharged.

## Discussion and conclusion

These two cases had no obvious causes and were considered idiopathic myelitis. Acute myelitis is a clinically transverse injury of the spinal cord, with symmetric limb weakness, symmetric sensory disturbances below the damaged segment and defecation disorders. However, few myelitis patients present with Brown-Séquard syndrome. A total of 22 studies were found on PubMed with the search term "Brown-Séquard syndrome and myelitis," of which nine studies indicated myelitis presenting as Brown-Séquard syndrome, as shown in our case report. Among the nine patients (male/female = 6:3) aged 13–53 years, the cause in four was diagnosed as viral infection, two as post-viral vaccination, two as idiopathic disease, and one as bacterial infection; five patients presented with the greatest damage in the thoracic segments and three in the cervical segments; one case was MRI-negative (Table 1).

**Table 1:** Information on the published case reports with "Brown-Séquard syndrome and myelitis"

Cases	Sex	Age (years)	Cause	Location
Abdul-Ghaffar 1988 [3]	Male	48	Virus	C5
Abdul-Ghaffar 1994 [4]	Male	13	Diphtheria and tetanus vaccinations	C4
Titlic 2006 [5]	Female	20	Meningococcal sepsis	T
Moon 2009 [6]	Male	38	Idiopathic	T11
Hosaka 2010 [7]	Female	32	Herpes zoster	T3-4
Vieira 2012 [8]	Female	52	H1N1 immunization	T
Dubey 2014 [9]	Male	53	CMV and AIDS	T5
Yamamoto 2019 [10]	Male	47	Idiopathic	C3-4
Elsayed 2019 [11]	Male	46	Varicella zoster	MRI (−)

*CMV Cytomegalovirus*, *AIDS* acquired immunodeficiency syndrome, *MRI* magnetic resonance imaging, *C* cervical, *T* thoracic

Case 1 presented as a long damaged segment with negative AQP4 antibody and was considered as NMOSD with negative AQP4 antibody. In accordance with the diagnostic criteria for NMOSD with negative AQP4 antibody, the patient presented with only one core clinical symptom of NMOSD. The patient also lacked other common symptoms of NMOSD, such as optic neuritis, posterior polar syndrome and brainstem syndrome [2]. If the patient had presented with other clinical symptoms or relapse, we could have diagnosed NMOSD. The onset in case 2 was typical of Brown-Séquard syndrome. However, the patient presented with weakness of both lower limbs, and MRI images only indicated lesions on the right side, indicating that the swelling caused by the lesions involved the left side or that the left lesion involved the left pyramidal tract, but this was not shown on MRI.

Brown-Séquard syndrome is caused by traumatic factors and nontraumatic factors. Traumatic factors are more common, for example, traffic accident injuries and blunt injuries, while nontraumatic factors include disk herniation, cervical spondylosis, tumors, multiple sclerosis, radioactive damage, decompression disease and spinal cord ischemia or bleeding. Infections also represent a nontraumatic factor, for example, tuberculosis, viruses including herpes zoster, bacterial infections and idiopathic transverse myelitis caused by infection-induced immune responses [1].

This case report demonstrates that myelitis can present as Brown-Séquard syndrome, providing an extended reference in terms of the differential diagnosis for clinical physicians.

## Data Availability

Not applicable.

## Abbreviations

CSF: Cerebrospinal fluid
MRI: Magnetic resonance imaging
OB: Oligoclonal band
AQP4: Aquaporin 4
CMV: *Cytomegalovirus*
EBV: Epstein-Barr virus
VEP: Visual evoked potential
NMOSD: Neuromyelitis optica spectrum disorders
